# Clinical and Differential Diagnosis in Typhoid Fever

**Published:** 1911-07

**Authors:** Cyrus W. Strickler

**Affiliations:** Atlanta, Ga.


### CLINICAL AND DIFFERENTIAL DIAGNOSIS IN TYPHOID FEVER.

By Cyrus W. Strickler, M.D., Atlanta, Ga.

The early diagnosis of typhoid fever is not nearly so easy as is sometimes considered. It is true that we may guess at the diagnosis and sometimes our suspicions may prove to be correct, but we may be easily fooled. The difficulties are made still more apparent when we remember the various clinical varieties we meet with. I need only refer to the ambulatory type, the afebrile type and the so-called cerebral typhoid. These may prove very puzzling for a time.

Thus it is evident that it is usually necessary to observe a case (just beginning) for some days before we can say with any degree of certainty the kind of fever with which we have to deal, and even after some days of observation and study we must of necessity complete our clinical data by the now well-known laboratory methods, which are too little used and respected.

Time will not permit me to take up the diagnosis of the various varieties to be met with clinically, so I will, therefore, discuss very briefly the more or less typical form.

An early diagnosis in this disease may usually be based upon the following evidence: the history of the patient, particularly if he has been a resident in a house where another patient has been suffering with this disease, or in a locality where the disease prevails, or if he takes his milk from the same milkman as a neighbor who is suffering with this malady. A negative history of a previous attack is considered by some to be helpful as a diagnostic point. I believe, however, that this is not considered as trustworthy as in the past.

Severe frontal, persistent headache resisting the usual remedies, particularly if accompanied by epistaxis, a coated tongue, moderately dilated pupils, dull expression, flushed face and malaise.

When the temperature curve is typical we get the gradual increase from day to day. This is more or less diagnostic. Another thing that doubtless you all have noticed is the fact that usually the tub or sponge baths fail to appreciably reduce this temperature for at least week or ten days. Another thing of some diagnostic value is the fact that a very small dose ot asperin will markedly lower the temperature of a typhoid and have little or no effect upon any other temperature I know of. I believe this statement is to some extent correct, and I have used it more than once as an aid in diagnosis. Only recently I so obscured a tonsillo-typhoid until the diagnosis was forced upon me by the clinical course of the case.

A diagnostic point of much value is the relationship between the pulse and the temperature. The pulse is usually very slow in comparison to the height of the temperature and very frequently becomes dicrotic early in the disease.

A slight cough is nearly always present and upon physical examination evidences of a mild bronchitis may be found present. The respirations are usually slightly increased. Even at this early stage the typical odor of these patients may be noticed.

Chilliness is very frequently complained of, as a matter of fact the disease may be ushered in by a chill or these patients may have distinct rigors at stated intervals so resembling malaria that quinine has been given in large doses without effect. A blood examination will usually promptly settle the question and should be made in all instances where it is possible, because as has already been pointed out by competent observers, quinine tends to produce hemorrhage, and, therefore, should not be given. Gastric symptoms, such as anorexia and slight nausea, are frequently present and sometimes the disease is ushered in with attacks of nausea and vomiting which may last for a number of days.

Sometimes there is severe pain and muscle spasm on the whole of the right side of the abdomen, particularly marked in the upper right quadrant, thus making a differential diagnosis between an acutely inflamed appendix or gall bladder necessary. Usually, however, there is only a slight amount of tenderness over the abdomen, a little more marked over the lower ileum. An increased amount of gas is usually present, particularly over the caecum and ascending colon.

An examination of the blood has shown that a leukopaenia is nearly always present and is a diagnostic point of considerable value. After a few days the patients become more or less apathetic.

At the end of a week or ten days the diagnosis is usually made without difficulty, for then in the usual run of cases we have the typical temperature curve, the large spleen and rose spots to make up so perfect a clinical picture that one could hardly fail to recognize this disease. But even if the rose spots be absent and we are unable to palpate the spleen the laboratory findings to which Dr. Paullin will call your attention will usually complete the picture.

Most writers upon the subject state that these patients suffer with a diarrhoea, as a matter of fact in the Spanish-American war many of these cases were considered as dysentery until the clinical course of the disease showed the error of this diagnosis. My own experience has been that the majority of these cases are constipated. I have seen some post-mortems on cases with very severe and extensive ulceration, the condition that usually produces a diarrhoea, yet they were constipated through the course of the disease.

### Differential Diagnosis.

In a differential diagnosis I will call your attention first to the fact that as above stated this disease may be mistaken for an attack of dysentery. A proper examination of the patient's blood and excreta is usually sufficient to enable us to come to correct conclusions.

There is now little excuse for either mistaking typhoid for malaria or giving quinine to make a diagnosis, as Dr. Paullin will show in a few minutes.

A cerebral typhoid may easily be mistaken for meningitis, but even in the absence of the usual evidencee the white cell and differential white cell count and examination of the spinal fluid will enable us to differentiate the two. I have already referred to this disease being mistaken for acute inflammation of the gall bladder or appendix. Here in addition to the physical examination the laboratory methods will be found to be of the greatest service.

As one of the most frequent diseases requiring a differential diagnosis I would mention acute miliary tuberculosis and will give briefly the differential diagnostic points mentioned by Anders and Boston in their book on Medical Diagnosis, viz: *Previous* history of cough, exposure to tubercular infection, tuberculous ulcer, abscess or fistula.

*Fever* rises more abruptly and remains high until death.

*Respirations* are very rapid, cyanosis to a greater or less degree is usually present, and upon examination of the chest physical signs of this condition may or may not be found.

The cough usually increases and the expectoration may be tinged with blood. Smears of the sputum may show the presence of the tubercle bacillus.

Constipation is the rule.

Rose spots are absent.

Wedal negative.

Instead of the typhoid bacillus being demonstrated in the stool tubercle bacilli may be found.

I thnk subacute miliary tuberculosis is very frequently mistaken for this disease. Several such cases, so regarded have come under my observation. I once treated such a case for typhoid fever for some days before finding out my error. When we remember that many of these cases have few or no physical signs, you can readily understand how we might be led astray for a time. We must rely upon the absence of an enlarged spleen, rose spots and laboratory methods to differentiate the two. The pulse in subacute miliary tuberculosis is either in keeping with the temperature or as is usually the case, more rapid than would be expected.

Candler Building, Atlanta, Ga.

